# Management of a Femur Shaft Fracture With Nancy Nail in the Setting of Dystrophic Epidermolysis Bullosa: A Case Report

**DOI:** 10.7759/cureus.21185

**Published:** 2022-01-12

**Authors:** Mohammed M Tarabishi, Shahd Almonaie, Mohamed Taha A Mohamed, Weam F Mousa

**Affiliations:** 1 Department of Orthopedic Surgery, King Fahad Medical City, Riyadh, SAU; 2 Department of Orthopaedic Surgery, Alfaisal University College of Medicine, Riyadh, SAU; 3 Department of Orthopedic Surgery, King Fahad Hospital, Madinah, SAU

**Keywords:** fracture, femur, nancy nail, orthopedic, epidermolysis bullosa

## Abstract

Management of bone fractures must achieve both reduction and stability. However, dermatological conditions such as dystrophic epidermolysis bullosa can lead to catastrophic events when operating on the patient’s bone fracture, possibly leading to wound infections and fracture nonunion.

Here, we report the case of a 20-year-old female with dystrophic epidermolysis bullosa who had suffered from a femur fracture after a fall from the bed. The fracture management was challenging due to the severe condition; however, the use of the Nancy nail was efficient.

Due to the rarity of the disease, modifications due to the challenges faced during the patient care approach were accomplished to prevent any harm to the patient. Even though the management was challenging, the outcome was good.

## Introduction

Children who suffer from epidermolysis bullosa are often referred to as “Butterfly children” as their skin is as vulnerable as the wings of a butterfly [1]. Epidermolysis bullosa is an entity of heterogenous bullous dermatoses including cutaneous fragility leading to bullous formation after exposure to trauma despite how minimal it is. Dystrophic epidermolysis bullosa (DEB) is a rare inherited form of the disease, characterized by the formation of bullae that further lead to atrophic scars [2-7]. The prevalence of DEB is 2-6 affected individuals per million births. The symptoms of DEB start at birth [3]. Type VII collagen is responsible for the formation of dermal-epidermal adhesion that remains stable as it is one of the building blocks of the anchoring fibrils; however, this is altered in DEB [4-7].

## Case presentation

A 20-year-old female with a history of DEB presented to the emergency room due to a fall from the bed. On initial examination, the patient was conscious with a Glasgow Coma Scale score of 15/15, pale, and cachectic. The patient’s appearance was not suitable for her current chronological age. She was vitally hypotensive in a third-grade hypovolemic shock. The Advanced Trauma Life Support protocol was initiated and 2 L of Ringer lactate was administered along with morphine even though intravenous access was difficult. Afterward, the patient moved to a state of transient normotensive shock. Severe pain and an obvious deformity of the right femur were notable with observable wounds or new skin blisters.

Her medical history was positive for DEB that was followed up by a dermatologist. Dilated cardiomyopathy, anal fissures, severe malnutrition, and iron deficiency anemia were present. Additionally, the patient complained of abdominal cramps, constipation, epistaxis, difficulty swallowing, adhesive tight tongue, unexplained shortness of breath, and dental caries. Although surgical and trauma history was unremarkable, a hospital admission 10 years ago due to shortness of breath was reported. At that time, three units of blood were administered due to a hemoglobin level of 5 g/dL.

The family history was positive for DEB in two siblings. On examination, scoliosis was evident along with right thigh deformity and flexion contracture of both hip joints of approximately 20 degrees. Both knees showed flexion contracture of approximately 40 degrees, with both feet being equinus and displaying a “mitten” appearance. The right thigh had scattered healed wounds due to the skin condition from all proximolateral aspects up to the middle region of the thigh. The distal thigh skin had several unhealed wounds all over the limb aspects. The range of motion in both lower limbs was normal up to the contractures. On an examination of the hands, flexed syndactyly due to the old skin blisters, wounds, and mitten appearance were noted. The distal pulses were intact, and the neurological examination was unremarkable.

**Figure 1:**
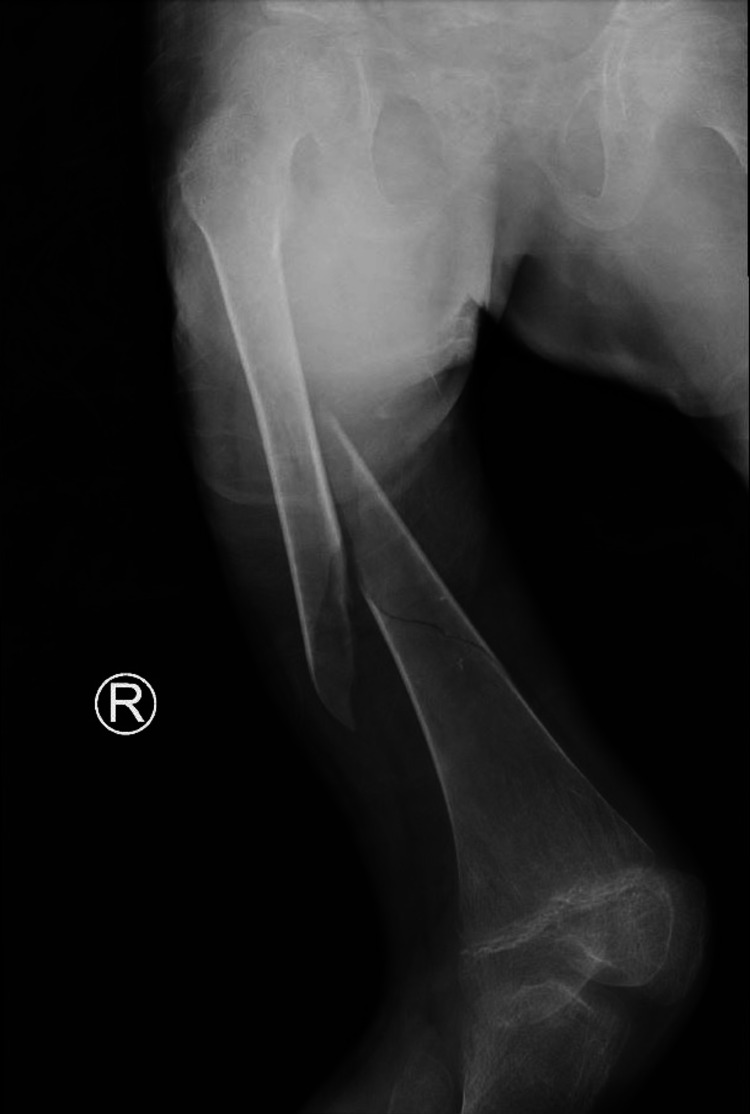
X-ray image taken on the day of the admission showing the hip, femur, and knee of a skeletally immature patient. A right femur spiral fracture in the mid-diaphyseal region can be seen along with a deformity that cannot be reduced due to epidermolysis bullosa and the fear of sloughing off the skin due to the nature of the disease. Narcotics and analgesia were administered and the patient was left in the best-preferred position for maximum comfort.

**Figure 2:**
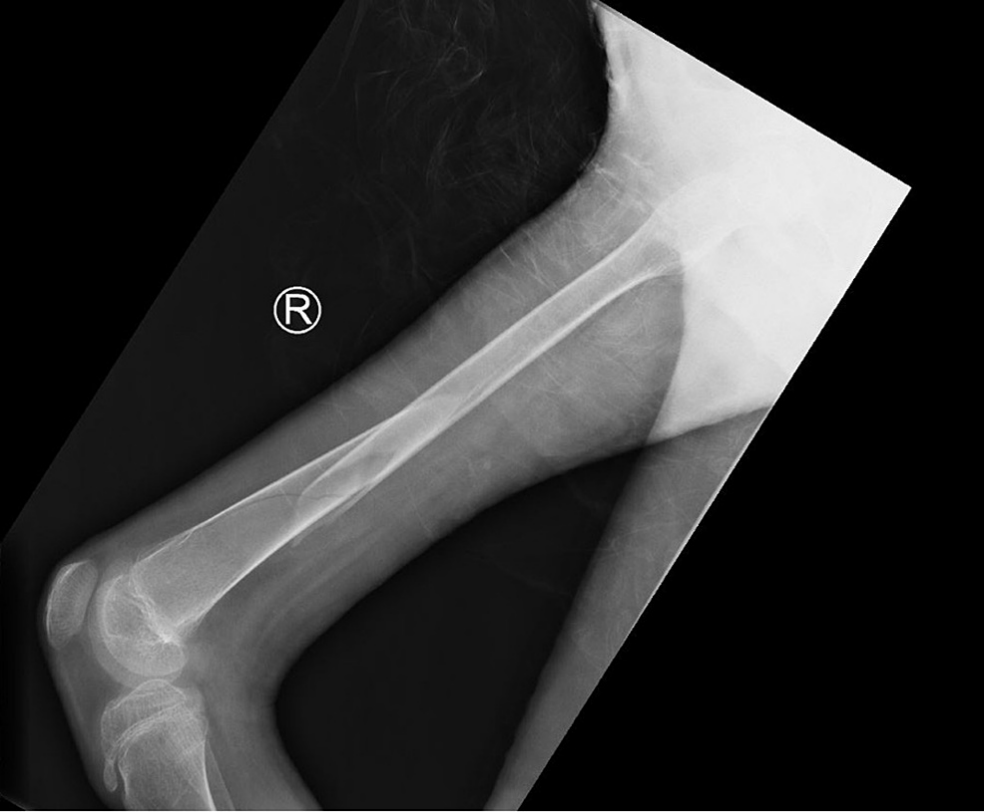
Lateral X-ray image taken on the day of the admission. Fracture of the femur and a crack in the distal diaphyseal region can be seen. However, the crack shown is not a fracture.

**Figure 3:**
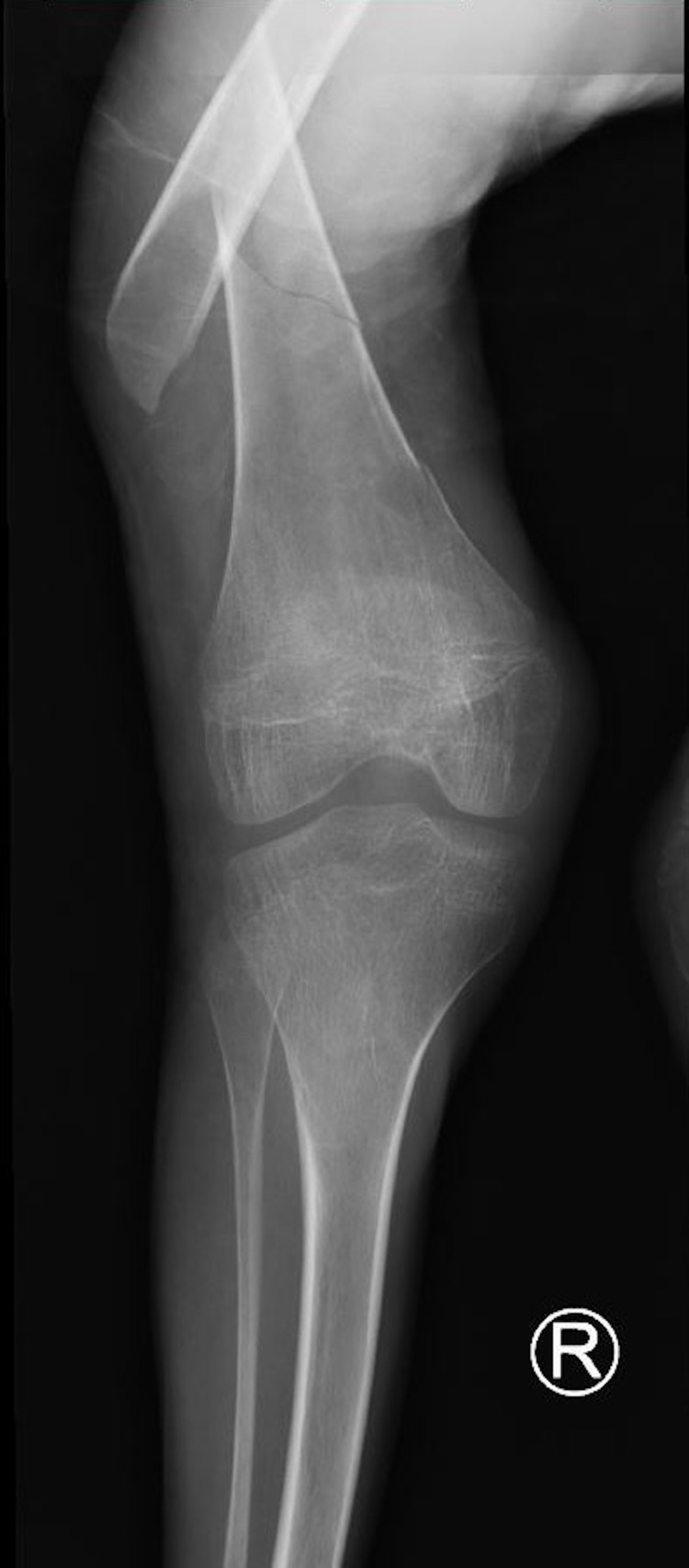
An X-ray image of the knee showing the knee joint and the distal femur physis which has not fused.

**Figure 4:**
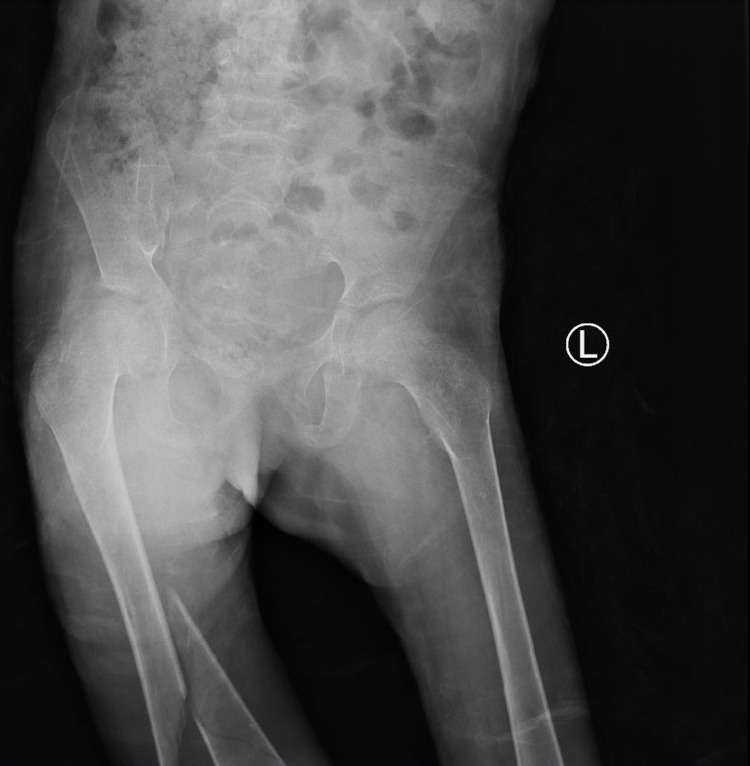
An X-ray image of the knee showing the knee joint and the distal femur physis which has not fused.

Anteroposterior and lateral views of the pelvis and right hip X-rays were obtained demonstrating a femur spiral mid-diaphyseal fracture (Figures 1-4).

Routine complete blood count showed iron deficiency anemia with a hemoglobin level of 2.5 g/dL and albumin of 7 g/L. Due to the medical history of dilated cardiomyopathy and in preparation for the management, an echocardiogram was obtained which showed an ejection fraction of 48% along with a systolic murmur.

The patient was admitted to the in-patient ward where she received three units of whole blood and albumin. Due to the lack of information available regarding the treatment of femur fracture in a case complicated by DEB, the team discussed the case thoroughly to approach the patient using a method that does not harm the skin and save the patient at any cost. However, the team found that the most suitable surgical treatment in this setting is the use of Nancy nail, which is a flexible intramedullary nail with the aid of a Steinmann pin in the closed versus open reduction and internal fixation for the femur fracture. The open reduction was not a possible treatment option due to DEB. We selected this treatment approach to avoid traumatizing the skin with countertraction, decrease the potential of infection, and reduce the risk of skin sloughing. The team discussed the plan with the patient and her family listing all the pros and cons as well as the possible complications. Consent was given to conduct the operation along with an agreement to hospitalize the patient three days prior to the day of the surgery for preparation.

**Figure 5:**
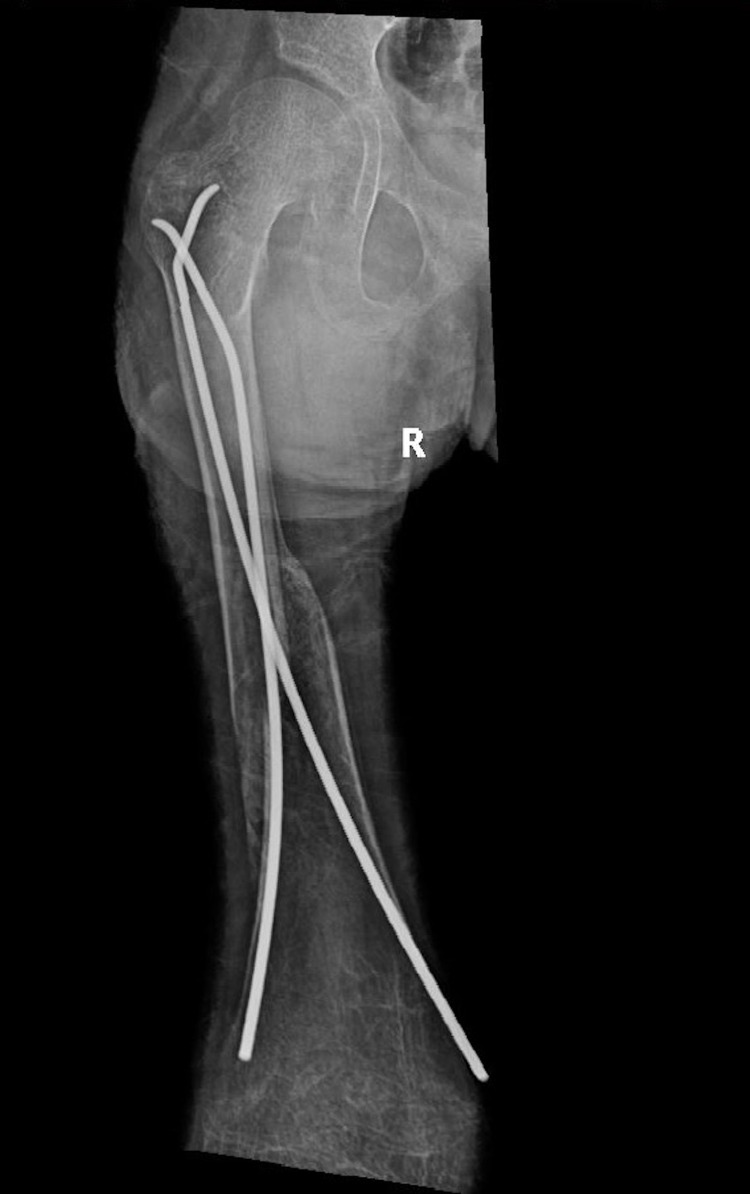
Anteroposterior view of an X-ray image of the femur eight months postoperatively with delayed healing of the femur. The Steinnman pin in the reduction with the use of the Nancy nail can be seen. There is a translation in the distal mechanical access of the femur with a 2 cm shortening because of the closed technique. Complete bone healing was established 18 weeks after the operation.

**Figure 6:**
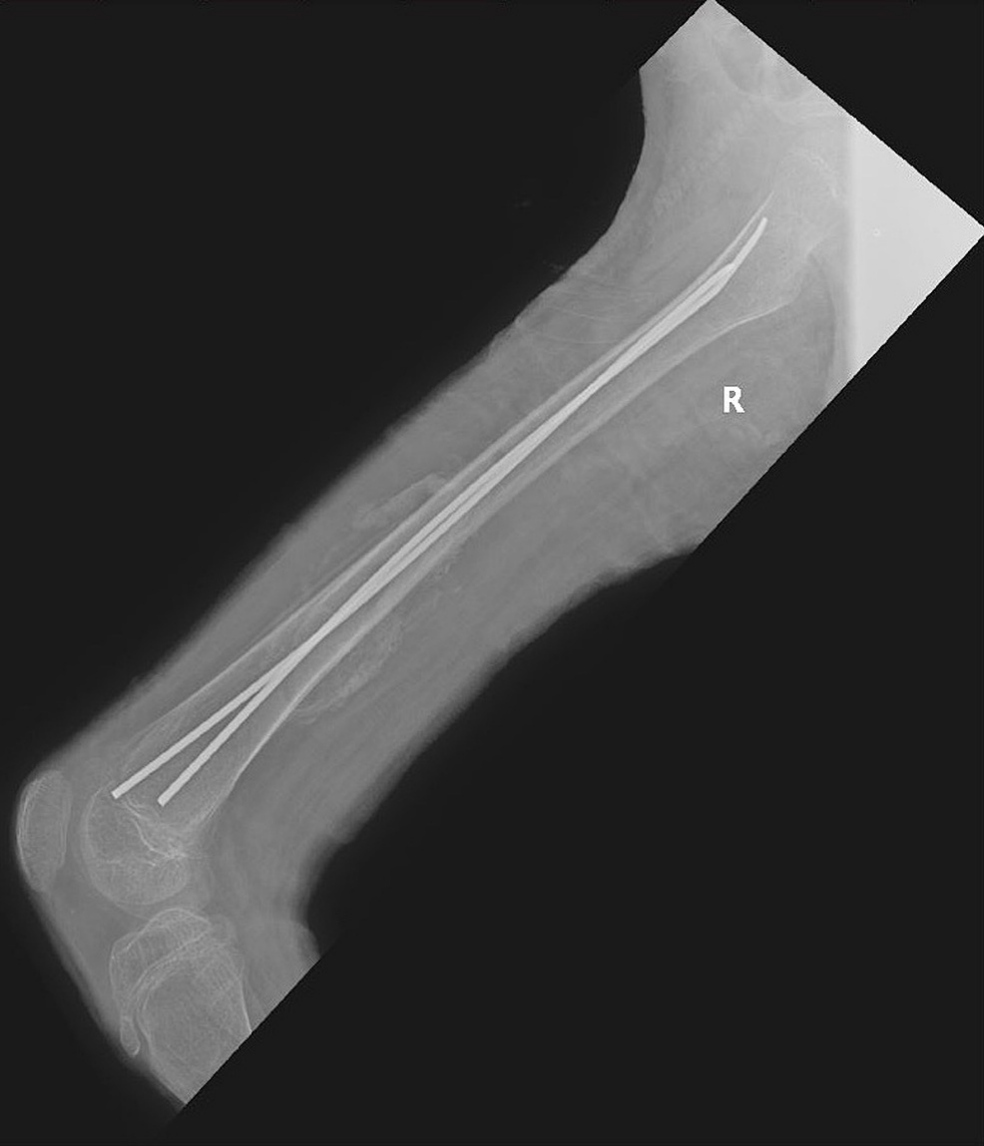
Lateral X-ray image showing acceptable sagittal reduction with good bone healing.

The patient had a Mallampati score of 4 and was at risk for esophageal perforation and difficult general intubation. The team agreed to perform spinal anesthesia and admit the patient to the postoperative intensive care unit. After prepping and draping, we made two 3 cm longitudinal incisions in the distal medial and lateral right distal thigh. Subsequently, 2.5 mm and 3 mm Nancy nails were introduced along with a Steinmann pin during the closed reduction procedure. The layers were closed with Vicryl sutures followed by wet dressing. The team faced the challenge of dealing with fragile bone during the operation as all the steps had to be performed very delicately. However, with the option of Nancy nail, the team could manage the fracture. The patient was admitted to the intensive care unit for close monitoring for one day followed by two days in the burn unit. Along with skin dressing, antibiotics, analgesics, and parenteral feeding with electrolytes and albumin correction were done. The patient was discharged home without any complications, and the family was informed about all the needed considerations to care for the patient. The wound healed with no ulcers, sinus tracts, or infection around the surgical wound (Figures 5, 6).

**Figure 7:**
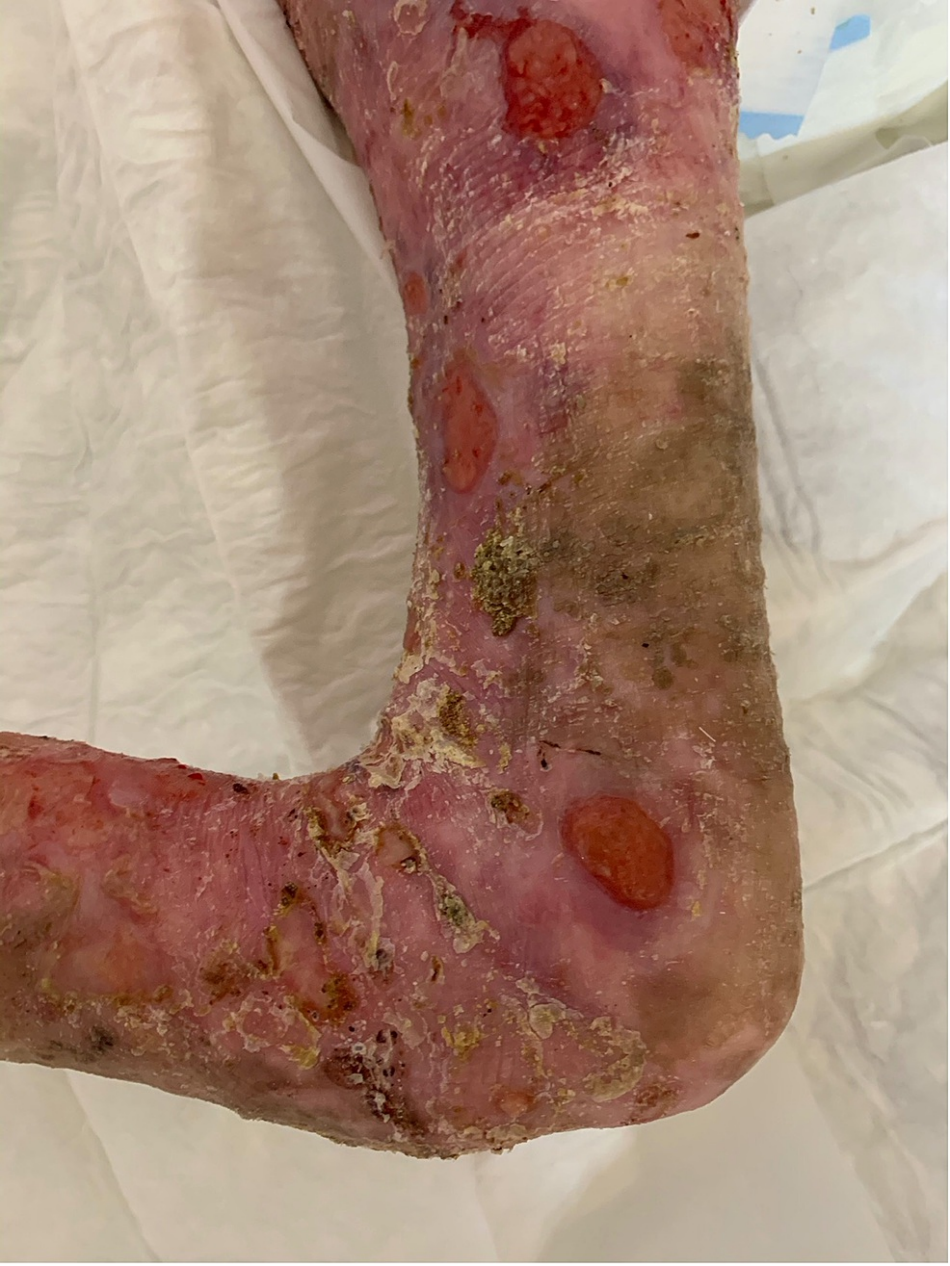
Postoperative image of the lateral aspect of the thigh.

**Figure 8:**
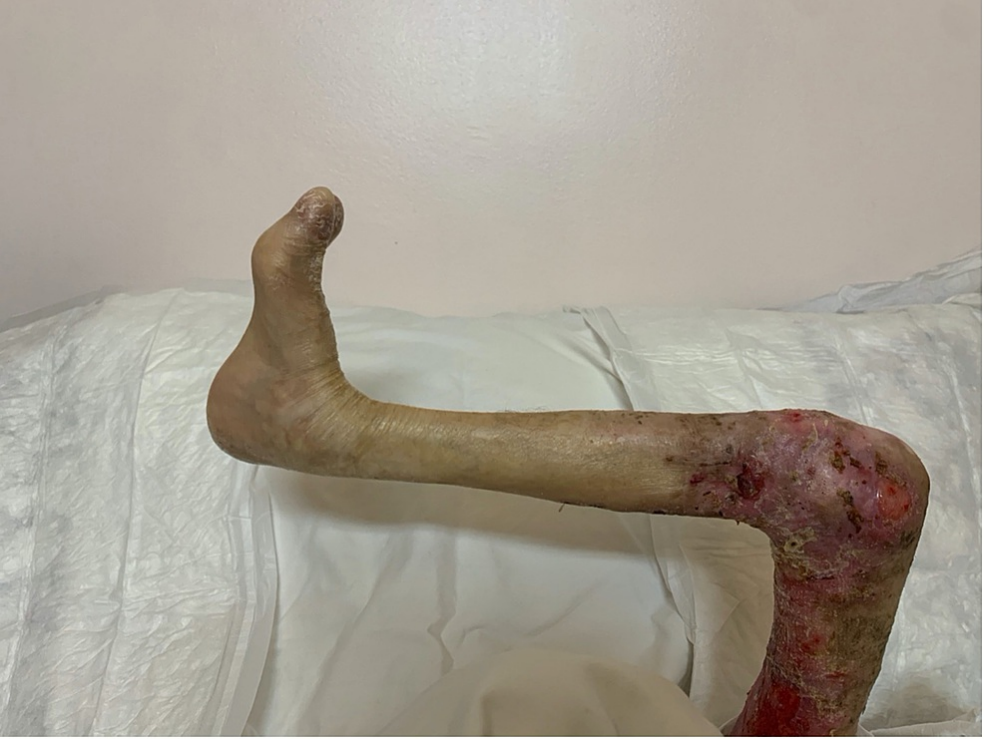
Postoperative image of the medial aspect of the thigh.

Two months postoperatively, the patient was seen for follow-up; an X-ray was done that showed an ingoing healing femur that was incomplete, with complete healing established only after 18 weeks (Figures 7, 8).

Although the rehabilitation program was implemented, the patient did not gain a weight-bearing status until after eight months of rehabilitation, including range of motion, stretching, and strengthening exercises. She was incapable of weight-bearing because of the pain and contracture of the knee and Achilles tendon. The patient started walking with walking aids approximately four months postoperatively, but she did not gain complete weight-bearing due to a decrease in the range of motion compared to her previous state. The patient may need another operation to correct her femur mechanical axis because she gained a deformity that affected her ability to walk. This deformity occurred because of the fracture pattern and the deforming force of the thigh muscle. The aim was to regain the length, correct the rotation, and prevent sagittal and coronal deformity as much as possible. However, we did not achieve the anatomical reduction because the team wanted to prevent further traumatic injuries to the soft tissue in the form of sloughing to the skin as mechanical trauma or causing an iatrogenic fracture. Factors such as the physis not being completely fused, body weight, and skin condition affected the decision of the surgery type and the instruments chosen. A plate and screw with closed reduction could not be used due to the open approach in the lateral thigh in the presence of scars and blisters increasing the risk of infection and delayed wound healing. The diameter of the canal was very narrow justifying the use of the Nancy nail. In choosing the best treatment modality, we considered that we need an acceptable reduction and initiate rapid rehabilitation. During the preoperative, operative, and postoperative phases of treatment, the team handled the case carefully. The soft tissue in the area of the fracture was well taken care of despite the critical skin condition.

## Discussion

The patterns of inheritance associated with DEB are autosomal dominant and recessive. The dominant type often manifests at birth or during early childhood and is characterized by generalized blistering that becomes more localized with age. The recessive type ranges in severity from mild to the severe form. Feet and hands pseudosyndactyly, referred to as “boxing glove hands,” deformed nails and affected teeth, flexural contractures, and iron deficiency anemia are all manifestations of the condition [5]. Scar tissue formation is often the sequelae of chronic skin ulcers that occur in a recurrent cycle and can lead to major dysfunction [3]. The management of DEB is challenging, and no curative treatment is currently available [6]. In 1968, Becker and Swinyard [8] were the first to find the association between DEB and reduced long bone mineralization as well as the manifestation of dental caries in four cases of DEB in children. This, in turn, increases the risk of developing osteopenia, osteoporosis, and pathological fractures [7]. Reduced intake and absorption of calcium decrease the overall production of vitamin D, malnutrition, reduced body mass index, and a history of a fracture event, leading to an increased risk of osteoporosis in DEB [9]. Low hemoglobin levels found in DEB patients and reduced plasma albumin are factors that increase the potential risk of low bone mass [10]. Unfortunately, there is a lack of information regarding the management of osteoporosis in the setting of DEB [11]. For the femur spiral fracture management in our case, we chose to use Nancy nail, which is a flexible intramedullary nail, along with the Steinmann pin in the closed versus open reduction and internal fixation for the femur fracture. We chose this approach after a thorough literature review, concluding that an open reduction was a possibly harmful option due to the increased risk of infection and complications. Aksoy et al. [12] reported that flexible intramedullary nailing is associated with a shorter intraoperative duration and a quicker healing process. Introducing this nail requires only small incisions [12]. The small incisions needed to introduce the nail are an advantage we needed in our patient to preserve the skin and avoid further harm. Moreover, the postoperative period of immobilization related to the use of this nail is shorter [12]. This key point is a considerable benefit in facing postoperative challenges. Lohiya et al. [13] have reported that flexible intramedullary nailing reduces infection risk and promotes bone healing while being inserted in a closed technique.

## Conclusions

A femur fracture in the setting of DEB is challenging to manage. However, with proper decisions taken based on the patient condition and a thorough literature review, optimum management can be sought. In our case, the use of Nancy nail was efficient, and the overall management led to good outcomes.

## References

[1] Prasad AN (2011). Epidermolysis bullosae. Med J Armed Forces India.

[2] Abboud L, Leclerc-Mercier S, Bodemer C, Guéro S (2021). Hand surgery in recessive dystrophic epidermolysis bullosa: our experience with dermal substitutes [In Press]. J Plast Reconstr Aesthet Surg.

[3] Shinkuma S (2015). Dystrophic epidermolysis bullosa: a review. Clin Cosmet Investig Dermatol.

[4] Shinkuma S, McMillan JR, Shimizu H (2011). Ultrastructure and molecular pathogenesis of epidermolysis bullosa. Clin Dermatol.

[5] Boeira VL, Souza ES, Rocha Bde O, Oliveira PD, Oliveira Mde F, Rêgo VR, Follador I (2013). Inherited epidermolysis bullosa: clinical and therapeutic aspects. An Bras Dermatol.

[6] Rashidghamat E, McGrath JA (2017). Novel and emerging therapies in the treatment of recessive dystrophic epidermolysis bullosa. Intractable Rare Dis Res.

[7] Chen JS, Yang A, Murrell DF (2019). Prevalence and pathogenesis of osteopenia and osteoporosis in epidermolysis bullosa: an evidence-based review. Exp Dermatol.

[8] Becker MH, Swinyard CA (1968). Epidermolysis bullosa dystrophica in children. Radiologic manifestations. Radiology.

[9] Hubbard LD, Mayre-Chilton K (2019). Retrospective longitudinal study of osteoporosis in adults with recessive dystrophic epidermolysis bullosa. Clin Case Rep.

[10] Giannoudis VP, Panteli M, Aderinto J, Giannoudis PV (2021). Reverse oblique proximal femoral fracture in dystrophic epidermolysis bullosa: challenges and recommendations. BMJ Case Rep.

[11] Kim M, Yang A, Murrell DF (2016). [Epidermolysis bullosa - why does a multidisciplinary team approach matter?]. Turk J Dematol.

[12] Aksoy C, Çaolar Ö, Yazycy M, Surat A (2018). Pediatric femoral fractures: a comparison of compression-plate fixation and flexible intramedullary nail fixation. Orthop Proceed.

[13] Lohiya R, Bachhal V, Khan U (2011). Flexible intramedullary nailing in paediatric femoral fractures. A report of 73 cases. J Orthop Surg Res.

